# Burned-Out: Middle School Teachers After One Year of Online Remote Teaching During COVID-19

**DOI:** 10.3389/fpsyg.2022.802520

**Published:** 2022-03-10

**Authors:** Tony Gutentag, Christa S. C. Asterhan

**Affiliations:** Seymour Fox School of Education, The Hebrew University of Jerusalem, Jerusalem, Israel

**Keywords:** burnout, wellbeing, COVID-19, online, remote teaching, online teaching proficiency, family work conflict

## Abstract

During the height of the COVID-19 pandemic, teachers around the globe had been forced to move their teaching to full-time online, remote teaching. In this study, we aimed at understanding teacher burnout during COVID-19. We conducted a survey among 399 teachers at the peak of a prolonged physical school closure. Teachers reported experiencing more burnout during (vs. before) the COVID-19 pandemic. Contributing factors to this burnout were high family work conflict and low online teaching proficiency. Burnout was associated with lower work-related wellbeing: Lower work commitment, and higher turnover intentions. It was also associated with lower psychological wellbeing: More depressive and anxiety symptoms, and lower subjective wellbeing. Approach (but not avoid) coping strategies served as a protective factor against the burnout-turnover intentions association. We conclude with recommendations on how to mitigate teacher burnout, thereby contributing to teacher wellbeing.

## Introduction

The spread of COVID-19 caused unprecedented interruptions to education worldwide. These affected students’ wellbeing (e.g., Radwan et al., 2020), their scholastic outcomes, and their financial future (Azevedo et al., 2021). The pandemic also affected their teachers, who were forced to adapt overnight to teaching from home in full online, remote formats. In the present investigation, we explore middle school teachers’ burnout, when they had been teaching in online, remote formats for over a year, due to pandemic-related physical school closures. We examined whether they experienced more burnout during (vs. before) the pandemic, explored possible contributing factors to burnout, and its associations with their wellbeing. Finally, we tested possible protective factors against the burnout-wellbeing association.

### Teacher Burnout

Burnout is characterized by emotional exhaustion, depersonalization of students, and low levels of personal accomplishment (Maslach and Jackson, 1981). Teacher burnout is negatively associated with student motivation (Shen et al., 2015), and student academic success (Madigan and Kim, 2021). A significant proportion of secondary school teachers suffer from high levels of burnout (García-Carmona et al., 2019). The COVID-19 pandemic and the subsequent social restrictions introduced additional stressors to the already high demanding profession of teaching. A survey conducted at the beginning of the pandemic, found a high level of teacher burnout (Pressley, 2021). It is therefore important to understand teacher burnout during COVID-19 pandemic, its extent, contributing factors, correlates, and the protective factors against it.

### Contributing Factors to Teacher Burnout

The COVID-19 related restrictions introduced two significant changes to the teaching profession that occurred overnight and lasted for prolonged periods of time: Teaching from home (instead of in designated spaces, that is: schools) and teaching using remote, online communication technologies (instead of face-to-face and collocated). Therefore, a first contributing factor to teacher burnout could be increased family work conflict especially during lockdowns. Teachers were forced to teach from their private homes, while also caring for other family members (e.g., children, elderly) and juggling between family and work duties. A recent study among healthcare workers during COVID-19 showed that family work conflict was one of the contributing factors to burnout (Cotel et al., 2021). A second contributing factor to increased teacher burnout during the pandemic could be the conversion to full-time online, remote teaching. Previous work has shown that online instructors suffer from more burnout in general (Hogan and McKnight, 2007). During the pandemic, teachers mentioned the absence of preparation, training and proper infrastructure as a major challenge of online, remote teaching during the pandemic (Kundu and Bej, 2021).

### Correlates of Teacher Burnout

Burnout goes hand-in-hand with teacher wellbeing (e.g., Tiwari et al., 2020). Substantial increases in distress were reported in the first months of the pandemic (Daly and Robinson, 2021), with over two-third of teachers reporting that the pandemic had negatively affected their psychological health (Allen et al., 2020). With respect to work-related wellbeing, burned-out teachers are less committed to their jobs, and consider leaving the profession more often (Ford et al., 2019). With respect to psychological wellbeing, burned-out teachers also experience more depression and anxiety (Schonfeld and Bianchi, 2016), and lower subjective wellbeing (Tiwari et al., 2020).

### Protective Factors Against Teacher Burnout

Despite the harmful effects that the COVID-19 pandemic may have had on teacher wellbeing, there may also be protective factors moderating the burnout-wellbeing association during this time. Ways to cope with stressful life events can be divided into approach (functional; e.g., acceptance) and avoid (dysfunctional; e.g., denial) strategies (Carver and Scheier, 1998). A survey study conducted among teachers during the beginning of the conversion to online remote teaching, found that approach, but not avoid, coping strategies were associated with better psychological outcomes (i.e., wellbeing, health, happiness, resilience, growth during trauma; MacIntyre et al., 2020).

### The Present Investigation

Since the inception of the COVID-19 pandemic, there were three state-mandated lockdowns in Israel. The exit strategy from each lockdown involved several steps, yet mainstream middle schools were always the last to return to face-to-face, collocated teaching and learning. The data for this investigation was collected toward the end of the third lockdown in Israel (February to March, 2021). We chose to focus on middle school teachers since at the time of data collection they were still mandated to teach in online, remote formats, and had been doing so for almost one full year.

Our research hypotheses in this investigation were as follows:

(H1) Middle school teachers experienced more burnout during (vs. before) the COVID-19 pandemic.

(H2) High family work conflict and low online teaching proficiency would be contributing factors to teacher burnout during the COVID-19 pandemic.

(H3) Greater teacher burnout during the COVID-19 pandemic would be associated with lower work-related and psychological wellbeing.

(H4) Approach, but not avoid, coping strategies would moderate the association between burnout and teacher wellbeing during the COVID-19 pandemic.

## Method

### Participants

The final sample consisted of 399 teachers (see Table 1 for demographics). The sample was representative with respect to the gender distribution of teachers in Israel (Israeli Central Bureau of Statistics, 2019), and social-economic ranking in Israel (Israeli Central Bureau of Statistics, 2021). Four hundred and twenty additional teachers did not pass the selection criteria, which were being an active middle school teacher in the Hebrew speaking public education sector, who presently teaches in online, remote formats only, has given consent to participate and has completed the survey; 266 additional teachers did not complete the survey. Participants were recruited *via* an initiated contact with every public secular and religious school in the Hebrew speaking sector of the Israeli education system, and *via* Facebook and Whatsapp, until quota (2/3 secular and 1/3 religious public schools, to represent their relative frequency in the population in Israel) was met. Participants were compensated with vouchers equivalent to 50 NIS.

**TABLE 1:** Teacher demographics.

Demographic	Statistics
Age	*M*_*age*_ = 38.01, *SD*_*age*_ = 10.41
Gender	76.2% female
Native Hebrew speakers	90.79%
Social-economic ranking	*M* = 6.18, *SD* = 1.87 (on a 1–10 scale)

A power analysis using G*Power 3.0 (Faul et al., 2007) indicated that a sample of 368 was required to detect a small-medium effect (*R*^2^ = .03), with 80% power, and .05 alpha. We increased this sample size by approximately 10% to account for potential attrition.

### Procedure

The study received approval from local research ethics committees.^1^ Data was collected between late February to early March, 2021, during the final weeks of the third lockdown in Israel, by which middle school teachers in Israel had been teaching in full online, remote conditions for the majority of the preceding year. The survey was conducted online and in Hebrew. Teachers read the general information and consented to participate anonymously. Then, they rated their online teaching proficiency, and family work conflict, burnout, job commitment, turnover intentions, depressive and anxiety symptoms, subjective wellbeing, coping strategies, and the change in their burnout compared to before the pandemic. Finally, they completed a demographic questionnaire.

### Materials

The survey included the following questionnaires (58 items in all in 10 questionnaires).

#### Change in Burnout

Teacher rated their work-related burnout during online, remote teaching, compared to before the COVID-19 pandemic (i.e., “my work-related burnout”; −3 = less than before the pandemic, 0 = the same, and 3 = more than before the pandemic).

#### Burnout

Teachers rated (1 = very mild, barely noticeable; 7 = very strong, major) the nine-item short-version of the Burnout scale (Maslach and Jackson, 1981; e.g., “I feel emotionally drained from my work.”; α = .80).

#### Online Teaching Proficiency

Teachers rated (1 = strongly disagree; 7 = strongly agree) the item: “I am successful in remote online teaching.”

#### Family Work Conflict

Teachers rated (1 = strongly disagree; 7 = strongly agree) the five-item family work conflict scale (Netemeyer et al., 1996; e.g., “Family related strain interferes with my ability to perform job related duties.”; α = .92).

#### Job Commitment

Teachers rated (1 = not at all; 5 = extremely) the four-item Klein et al. (2014), Unidimensional, Target-free (KUT) measure (e.g., “How committed are you to the teaching vocation?”; α = .91).

#### Turnover Intentions

Teachers rated (1 = certainly not, 4 = maybe, and 7 = certainly yes) the item: “Suppose you were starting your life over again, would you choose a teaching career again?” (Borg et al., 1991). We reverse-scored this item, so that higher scores indicated higher turnover intentions.

#### Depressive Symptoms

Teachers rated the frequency (1 = rarely or none of the time; 4 = most or all of the time) of two representative symptoms (“I felt depressed,” and “I could not get ‘going”’) from the short Center for Epidemiologic Studies Depression scale (CES–D; Radloff, 1977), referring to the past month (α = .77).

#### Anxiety Symptoms

Teachers rated the frequency (0 = not at all; 3 = nearly every day) of two representative symptoms (“Feeling nervous, anxious, or on edge,” and “Not being able to stop or control worrying”) from the seven-item brief Generalized Anxiety Disorder scale (GAD-7; Spitzer et al., 2006), referring to the past month (α = .86).

#### Subjective Wellbeing

Teachers rated (1 = strongly disagree; 7 = strongly agree) the five-item Satisfaction with Life Scale (Diener et al., 1985; e.g., “I am satisfied with my life.”; α = .87).

#### Approach Coping Strategies

Teachers rated (1 = I have not been doing this at all; 4 = I’ve been doing this a lot) the 28-item brief COPE (Carver, 1997). Items were grouped into two types of strategies, approach (“I’ve been trying to come up with a strategy about what to do.”; α = .81), and avoid (“I’ve been criticizing myself”; α = .70).

## Results

We conducted analyses in R (version 1.3.1093). Teachers reported experiencing more burnout during COVID-19 pandemic, compared to before the pandemic (one Sample’s *t*-test comparing teachers’ mean change in burnout ratings to 0, indicating no change; *t*(398) = 9.27, *p* < .001, *d* = 0.65).

To examine the possible explanatory factors of burnout, we conducted a multiple regression analysis. Family work conflict and online teaching proficiency served as predictors (independent variables) of burnout during COVID-19 online, remote teaching (dependent variable). The model significantly predicted burnout [*F*(2, 396) = 77.06, *p* < .001, Multiple *R*^2^ = .28], and explained 28% of its variance. Each of the predictors contributed significantly to the model, the strongest being family work conflict [*B* = 0.20 (*SD* = 0.02), β = .35, *t* = 8.00, *p* < .001], followed by online teaching proficiency [*B* = −0.27 (*SD* = 0.03), β = −.33, *t* = −7.61, *p* < .001].

Next, we examined the correlates of burnout. As can be seen in Table 2, higher burnout was associated with indicators of work-related wellbeing (i.e., lower work commitment, higher turnover intentions) and of psychological wellbeing (i.e., more depressive symptoms, more anxiety symptoms, lower subjective wellbeing).

**TABLE 2:** Means, standard deviations, and correlations of key variables (*N* = 399).

Variable	*Scale*	*M*	*SD*	1	2	3	4	5	6	7	8	9	10
1. Chang in burnout	(−3)–3	.75	1.62										
2. Burnout	1–7	2.95	.99	.22**									
3. Online teaching proficiency	1–7	5.38	1.23	–.09	−.40**								
4. Family work conflict	1–7	3.34	1.73	.24**	.42**	−.21**							
5. Job commitment	1–5	4.49	.63	.11*	−.42**	.37**	−.17**						
6. Turnover intentions	1–7	4.82	1.80	.05	.28**	–.05	.10	−.23**					
7. Depressive symptoms	1–4	2.08	.82	.29**	.53**	−.26**	.32**	−.26**	.20**				
8. Anxiety symptoms	0–3	1.13	.81	.30**	.50**	−.20**	.36**	−.15**	.17**	.60**			
9. Subjective wellbeing	1–7	5.03	1.22	–.08	−.32**	.22**	–.09	.37**	−.35**	−.33**	−.33**		
1. Approach coping strategies	1–4	3.05	.47	.09	−.28**	.19**	.01	.33**	−.22**	−.13*	–.09	.30**	
11. Avoid coping strategies	1–4	2.29	.43	.06	.33**	−.20**	.19**	−.20**	.04	.43**	.42**	−.26**	.10*

*p < .05, **p < .01.

**FIGURE 1:**
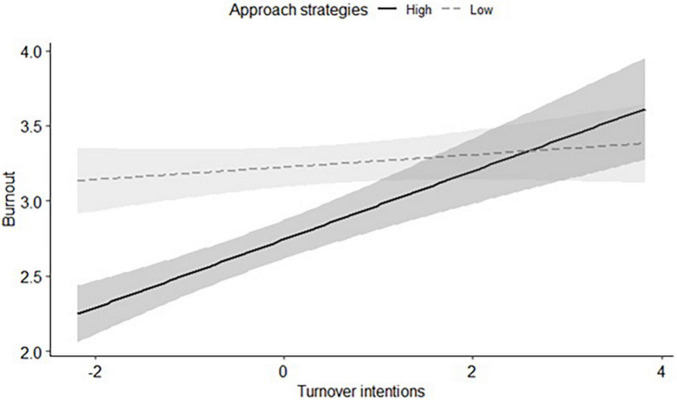
Burnout as a function of turnover intentions and approach coping strategies (means in the high and low groups are estimated based on ± 1 *SD* from the mean).

Finally, we examined coping strategies as a possible moderator against burnout. We examined approach and avoid coping strategies as moderator of the associations between burnout and work-related (i.e., work commitment, turnover intentions) and psychological wellbeing (i.e., depressive symptoms, anxiety symptoms, subjective wellbeing). After a Bonferroni correction for multiple comparisons (0.05/10 = .005), one significant moderation emerged, for approach coping strategies × turnover intentions interaction (*B* = 0.20, *SE* = 0.05, β = .17, *t* = 3.66, *p* < .001). There was a significant main effect for approach coping strategies (*B* = −0.50, *SE* = 0.10, β = −.24, *t* = −5.11, *p* < .001), and turnover intentions (*B* = 0.13, *SE* = 0.03, β = .24, *t* = 5.14, *p* = .001). As shown in Figure 1, among teachers lower in approach coping strategies, burnout was high regardless of turnover intentions level (*B* = 0.04, *SE* = 0.03, *t* = 1.20, *p* = .230); whereas among teachers higher in approach coping strategies, a positive association was found between burnout and turnover intentions (*B* = 0.23, *SE* = 0.04, *t* = 5.92, *p* < .001).^2^

## Discussion

After a stressful year, it is not surprising that teachers reported experiencing more burnout during (vs. before) the COVID-19 pandemic. Contributing factors to this burnout were high family work conflict, and low perceived proficiency for online teaching. The higher teacher burnout during COVID-19, the lower their work commitment is, and the higher their turnover intentions are. Higher teacher burnout was also associated with more depressive and anxiety symptoms, and lower subjective wellbeing. Approach coping strategies served as a protective factor against the association between burnout and turnover intentions, but not against the other associations of burnout (i.e., work commitment, depressive or anxiety symptoms, subjective wellbeing). As expected, avoid coping strategies did not serve as a protective factor against the burnout-wellbeing association.

### Theoretical and Pragmatic Implications

COVID-19 pandemic has taken its toll on teacher burnout (e.g., Pressley, 2021). Understanding teacher burnout is therefore important. The first contributing factors to teacher burnout was high family work conflict, which is consistent with previous research on healthcare workers (Cotel et al., 2021). Family work conflict was unrelated to gender (*r*_*PB*_ = −.04, *ns*), but was related to the mere fact of having kids (*r*_*PB*_ = .18, *p* < .001). Alleviating the family work conflict could therefore reduce chances of burnout. This can be achieved by, for example, offering subsidized childcare for working teachers, as was customary with respect to healthcare workers in many countries. The second contributing factors to teacher burnout was low online teaching proficiency. Promoting online teaching proficiency in general, and for teachers still struggling with ICT technologies one year into the pandemic in particular, seems a viable way to reduce teacher burnout.

High burnout was associated with low teacher wellbeing. In line with previous research conducted before the pandemic (Ford et al., 2019), teachers’ work-related wellbeing was associated with lower job commitment, and higher turnover intentions during COVID-19 pandemic. Also in line with previous research prior to COVID-19 (Schonfeld and Bianchi, 2016; Tiwari et al., 2020), teacher psychological health during the pandemic was associated with more depressive and anxiety symptoms, and lower subjective wellbeing. Therefore, attending to teachers’ burnout is paramount, as it is directly associated with their work-related and psychological wellbeing. It might be wise to detect early signs of teacher burnout, and provide these teachers with immediate support (for example, a “hot line” for teachers).

Finally, the ultimate negative outcome of burnout is turnover. In line with previous study (MacIntyre et al., 2020), we found that approach (but not avoid) coping strategies were related to better outcomes among teachers, by moderating the burnout-turnover intentions association. Therefore, to reduce turnover intentions among teachers due to COVID-19, it might be best to focus on supporting such coping strategies (that is: acceptance, emotional or instrumental support, positive reframing, active coping, and planning; Carver, 1997).

### Future Directions

We highlight four limitations of the present study. First, the correlational design we employed does not allow for causal inferences. For example, within the current set-up, we cannot discern whether anxiety leads to burnout, or vice versa. Some have suggested that a significant predictor for teacher burnout during COVID-19 is anxiety (Pressley, 2021). It might also be that burnout leads to anxiety (e.g., Koutsimani et al., 2019). Future research could examine the directionality of these effects using longitudinal designs. Second, in this investigation, we conducted a cross-sectional survey. This form of inquiry does not allow us to examine the underlying process leading to the observed associations. For instance, it would be interesting to study how low online teaching proficiency shapes teacher burnout. Third, we only examined teacher turnover intentions and not actual turnover. A recent United States teacher survey indicated that one of every four teachers was thinking of quitting their job, of whom 70% attributed these intentions to the pandemic (Steiner and Woo, 2021). A systematic follow-up study could examine the extent to which teachers fulfilled these intentions. Finally, in the present investigation we focused on teachers, but not on their students, whose wellbeing and scholastic achievement could have been affected by teacher burnout.

## Data Availability Statement

The datasets analyzed for this study can be found in the OSF: https://osf.io/c4emp/?view_only=e6d962567a8d43308fb4928ae33fc637.

## Ethics Statement

The studies involving human participants were reviewed and approved by the School of Education, The Hebrew University of Jerusalem, and the Israeli Ministry of Education. The participants provided their written informed consent to participate in this study.

## Author Contributions

TG and CA contributed to the design and implementation of the research, to the analysis of the results, and to the writing of the manuscript. Both authors contributed to the article and approved the submitted version.

## Conflict of Interest

The authors declare that the research was conducted in the absence of any commercial or financial relationships that could be construed as a potential conflict of interest.

## Publisher’s Note

All claims expressed in this article are solely those of the authors and do not necessarily represent those of their affiliated organizations, or those of the publisher, the editors and the reviewers. Any product that may be evaluated in this article, or claim that may be made by its manufacturer, is not guaranteed or endorsed by the publisher.

## Footnotes

1 Data were collected as part of a larger study designed to answer multiple research questions, and we only report on variables relevant to this project. The survey was developed to answer this investigation’s research questions, as well as the research questions of related projects. When assembling the survey, we first listed all variables of interest in this and the other research projects. Then, we tried to find well-established and validated tools to assess each variable. When such tools were lacking, we developed items (e.g., change in burnout) to assess them. Due to the rapid nature of COVID-19 pandemic research, we did not pilot test the survey before administrating it to the research sample.

2 A test of multicollinearity indicates that Tolerance = 0.96, and VIF = 1.05. According to Ho (2006), tolerance of 0.10\VIF greater than 10 merits further investigation, which is not the case here.

## References

[B1] Allen R. Jerrim J. Simms S. (2020). *How did the early stages of the COVID-19 pandemic affect teacher wellbeing? Centre for Education Policy and Equalising Opportunities (CEPEO) Working Paper.* 20–15. Available online at: https://EconPapers.repec.org/RePEc:ucl:cepeow:20-15 (Accessed November 9, 2021).

[B2] Azevedo J. P. Hasan A. Goldemberg D. Geven K. Iqbal S. A. (2021). Simulating the potential impacts of COVID-19 school closures on schooling and learning outcomes. A set of global estimates. *World Bank Res. Obser.* 36 1–40. 10.1080/00131911.2021.2007054

[B3] Borg M. G. Riding R. J. Falzon J. M. (1991). Stress in teaching: a study of occupational stress and its determinants, job satisfaction and career commitment among primary schoolteachers. *Educ. Psychol.* 11 59–75. 10.1080/0144341910110104

[B4] Carver C. S. (1997). You want to measure coping but your protocol’s too long: consider the Brief COPE. *Internat. J. Behav. Med.* 4 92–100. 10.1207/s15327558ijbm0401_6 16250744

[B5] Carver C. S. Scheier M. F. (1998). *On the self-regulation of behavior.* New York, NY: Cambridge University Press.

[B6] Cotel A. Golu F. Pantea Stoian A. Dimitriu M. Socea B. Cirstoveanu C. (2021). Predictors of burnout in healthcare workers during the COVID-19 pandemic. *Healthcare* 9 1–8. 10.3390/healthcare9030304 33803286 PMC8001536

[B7] Daly M. Robinson E. (2021). Psychological distress and adaptation to the COVID-19 crisis in the United States. *J. Psychiat. Res.* 136 603–609. 10.1016/j.jpsychires.2020.10.035 33138985 PMC7588823

[B8] Diener E. D. Emmons R. A. Larsen R. J. Griffin S. (1985). The satisfaction with life scale. *J. Personal. Assess.* 49 71–75. 10.1207/s15327752jpa4901_13 16367493

[B9] Faul F. Erdfelder E. Lang A. G. Buchner A. (2007). G*Power 3: a flexible statistical power analysis program for the social, behavioral, and biomedical sciences. *Behav. Res. Methods* 39 175–191. 10.3758/bf03193146 17695343

[B10] Ford T. G. Olsen J. Khojasteh J. Ware J. Urick A. (2019). The effects of leader support for teacher psychological needs on teacher burnout, commitment, and intent to leave. *J. Educ. Administr.* 57 615–634. 10.1108/jea-09-2018-0185

[B11] García-Carmona M. Marín M. D. Aguayo R. (2019). Burnout syndrome in secondary school teachers: a systematic review and meta-analysis. *Soc. Psychol. Educ.* 22 189–208. 10.3390/brainsci11091172 34573192 PMC8468121

[B12] Ho R. (2006). *Univariate and multivariate data analysis and interpretation with SPSS.* Boca Raton, FL: Taylor & Francis Group, 203–238.

[B13] Hogan R. L. McKnight M. A. (2007). Exploring burnout among university online instructors: an initial investigation. *Internet High. Educ.* 10 117–124. 10.1016/j.iheduc.2007.03.001

[B14] Israeli Central Bureau of Statistics (2019). *Statistical 177.* Retrieved from: https://www.cbs.gov.il/he/Statistical/hinuh_2018.pdf (accessed February 25, 2022).

[B15] Israeli Central Bureau of Statistics. (2021). *Social**-economic Ranking.* Retrieved from: https://www.cbs.gov.il/he/subjects/Pages/%D7%9E%D7%93%D7%93-%D7%97%D7%91%D7%A8%D7%AA%D7%99-%D7%9B%D7%9C%D7%9B%D7%9C%D7%99-%D7%A9%D7%9C-%D7%94%D7%A8%D7%A9%D7%95%D7%99%D7%95%D7%AA-%D7%94%D7%9E%D7%A7%D7%95%D7%9E%D7%99%D7%95%D7%AA.aspx (accessed February 25, 2022).

[B16] Klein H. J. Cooper J. T. Molloy J. C. Swanson J. A. (2014). The assessment of commitment: advantages of a unidimensional, target-free approach. *J. Appl. Psychol.* 99 222–238. 10.1037/a0034751 24188389

[B17] Koutsimani P. Montgomery A. Georganta K. (2019). The relationship between burnout, depression, and anxiety: a systematic review and meta-analysis. *Front.* P*sychol.* 10:1–19. 10.3389/fpsyg.2019.00284 30918490 PMC6424886

[B18] Kundu A. Bej T. (2021). COVID 19 response: an analysis of teachers’ perception on pedagogical successes and challenges of digital teaching practice during new normal. *Educ. Inf. Technol.* 26, 6879. 10.1007/s10639-021-10503-5 33897267 PMC8053079

[B19] MacIntyre P. D. Gregersen T. Mercer S. (2020). Language teachers’ coping strategies during the COVID-19 conversion to online teaching: Correlations with stress, wellbeing and negative emotions. *System* 94:102352.

[B20] Madigan D. J. Kim L. E. (2021). Does teacher burnout affect students? A systematic review of its association with academic achievement and student-reported outcomes. *Internat. J. Educ. Res.* 105:101714. 10.1016/j.ijer.2020.101714

[B21] Maslach C. Jackson S. E. (1981). The measurement of experienced burnout. *J. Org. Behav.* 2 99–113. 10.1002/job.4030020205

[B22] Netemeyer R. G. Boles J. S. McMurrian R. (1996). Development and validation of work–family conflict and family–work conflict scales. *J. Appl. Psychol.* 81 400–410. 10.1037/0021-9010.81.4.400

[B23] Pressley T. (2021). Factors contributing to teacher burnout during COVID-19. *Educ. Res.* 2021:0013189X211004138.

[B24] Radloff L. S. (1977). The CES-D scale: A self-report depression scale for research in the general population. *Appl. Psychol. Meas.* 1 385–401. 10.1177/014662167700100306 26918431

[B25] Radwan E. Radwan A. Radwan W. (2020). The Mental Health of School Students and the COVID-19 Pandemic. *Aquademia* 4:2. 10.29333/aquademia/8394

[B26] Schonfeld I. S. Bianchi R. (2016). Burnout and depression: two entities or one? *J. Clin. Psychol.* 72 22–37. 10.1002/jclp.22229 26451877

[B27] Shen B. McCaughtry N. Martin J. Garn A. Kulik N. Fahlman M. (2015). The relationship between teacher burnout and student motivation. *Br. J. Educ. Psychol.* 85 519–532. 10.1111/bjep.12089 26235820

[B28] Spitzer R. L. Kroenke K. Williams J. B. Löwe B. (2006). A brief measure for assessing generalized anxiety disorder: the GAD-7. *Archiv. Inter. Med.* 166 1092–1097. 10.1001/archinte.166.10.1092 16717171

[B29] Steiner E. D. Woo A. (2021). *Job-Related Stress Threatens the Teacher Supply: Key Findings from the 2021 State of the U.S. Teacher Survey.* Santa Monica, CA: RAND Corporation.

[B30] Tiwari A. Saraff S. Nair R. (2020). Impact of emotional labor on burnout and subjective well-being of female counselors and female teachers. *J. Psychosoc. Res.* 15 523–532. 10.32381/jpr.2020.15.02.14

